# Diagnostic value of cardiovascular biomarkers for cerebral–cardiac syndrome risk in acute ischemic stroke

**DOI:** 10.3389/fmed.2026.1824631

**Published:** 2026-07-08

**Authors:** Zegang Liu, Ying Zhao, Mei Wang, Chenwei Li, Xurong Zhu, Peirui Wang, Ke Wang

**Affiliations:** 1 Clinical Laboratory, Key Laboratory of Intelligent Diagnosis and Treatment for Major Diseases of Yongchuan District, Yongchuan People’s Hospital of Chongqing, Chongqing, China; 2 Clinical Laboratory, Yubei People's Hospital of Chongqing, Chongqing, China; 3 Department of Research and Development, Shanghai i-Reader Biotech Co., Ltd., Shanghai, China

**Keywords:** acute ischemic stroke, B-type natriuretic peptide, cardiac troponin I, cerebral-cardiac syndrome, diagnostic model, myoglobin

## Abstract

**Background:**

Cerebral–cardiac syndrome (CCS) is a complication commonly observed following an ischemic stroke, which results in increased morbidity and mortality. Timely diagnosis of CCS is of prognostic significance. This study investigates the significance of multiple cardiovascular biomarkers for CCS in patients with acute ischemic stroke (AIS).

**Methods:**

This study retrospectively analyzed 177 patients with AIS admitted to Yongchuan People’s Hospital of Chongqing from March 2022 to March 2023. Plasma levels of BNP (B-type natriuretic peptide), cTnI (cardiac Troponin I), Myo (myoglobin), CK-MB (creatine kinase isoenzyme), D-Dimer (DD), FDP (fibrin degradation product), and CRP (C-reactive protein) were measured. Multivariable Firth’s penalized logistic regression was used to identify independent risk factors for CCS, and receiver operating characteristic analysis and bootstrap validation were performed to evaluate the diagnostic performance.

**Results:**

CCS occurred in 97 patients (54.8%). BNP, DD, cTnI, Myo, and CK-MB were significantly elevated in the CCS group compared to the non-CCS group (all *p* < 0.001). After adjustment for covariates, age (OR = 1.047, *p* = 0.036), BNP (OR = 1.028, *p* < 0.001), and Myo (OR = 1.003, *p* = 0.048) remained as independent risk factors. The combined model incorporating age, BNP, and Myo yielded an area under the curve of 0.945 (95% CI: 0.914–0.977), with bootstrap-adjusted AUC = 0.941, indicating excellent discriminatory ability of the model and strong concordance with the clinical diagnosis.

**Conclusion:**

Age, BNP, and Myo constitute a robust diagnostic model for the early identification of CCS in AIS patients. This panel may facilitate timely risk stratification and intervention in the clinical management of CCS.

## Introduction

Cerebral–cardiac syndrome (CCS) refers to brain–heart interaction, also known as neurocardiogenic syndrome. Various cerebral injuries such as ischemic stroke (IS), intracerebral hemorrhage (ICH), subarachnoid hemorrhage (SAH), traumatic brain injury, and stress are evidenced to cause cardiac injuries or exacerbate preexisting heart disease, manifesting as arrhythmia, myocardial damage, myocardial ischemia, heart failure, and myocardial infarction (1, 2). IS remains the most common type of cerebral insult, accounting for approximately 87% of new stroke cases among the population aged 40 and above in China in 2020 (3). Recent studies further showed that CCS, occurring in 25–75% stroke patients, depending on different study designs and examining methods (4, 5), is a complication common in stroke, which has a rapid onset and progression. Earlier studies that CCS was more common in cerebrovascular disease (CVD) patients than in other neurological diseases (5). In the early phase of the disease, it can serve as a risk indicator for further deterioration of stroke. CCS significantly affects prognosis, morbidity, and mortality of stroke, and accounts for the second leading cause of death after CVD. Compared with non-CCS patients, the probability of death in patients with CCS is 2.7 times higher (4). Therefore, early diagnosis of CCS is particularly important for treatment of the disease. Despite growing evidence, the mechanisms and risk factors of CCS remain poorly understood and controversial. Moreover, clinical awareness of CCS in stroke patients remains insufficient. Although risk prediction scales have been developed, and ECG, echocardiography, and troponin measurements are widely available in acute stroke care, a comprehensive diagnostic procedure for CCS remains challenging due to the time-sensitive nature of revascularization therapies (6, 7). This study aimed to identify risk biomarkers for CCS in patients with acute ischemic stroke (AIS). This study evaluated the diagnostic value of cardiovascular biomarkers in 177 AIS patients admitted to Yongchuan People’s Hospital of Chongqing between March 2022 and March 2023, aiming to early identification of high-risk factors to facilitate timely intervention.

## Materials and methods

### Study population

This retrospective case–control study was conducted on 177 patients with AIS admitted to the neurology department of Yongchuan People’s Hospital of Chongqing from March 2022 to March 2023. The diagnostic criteria were based on the Chinese Guidelines for Diagnosis and Treatment of Acute Ischemic Stroke (8, 9). According to the occurrence of CCS during the course of the disease, the patients were divided into the CCS group and non-CCS group. The CCS diagnostic criteria (10) were as follows: (1) no prior history of heart disease, (2) confirmed diagnosis of AIS through CT or MRI, (3) presence of new-onset of cardiac manifestations within 72 h after stroke, meeting at least one of the following criteria: (a) arrhythmias (atrial fibrillation/flutter, premature ventricular contractions, premature atrial contractions, sinus tachycardia, sinus bradycardia, second-degree or higher atrioventricular block, new-onset bundle branch block, prolonged QTc interval); (b) electrocardiographic (ECG) changes (ST-segment elevation or depression, T-wave inversion or flattening, new Q waves); and (c) echocardiographic or clinical evidence suggesting cardiac dysfunction or myocardial ischemia. The B-type natriuretic peptide (BNP), cardiac Troponin I (cTnI), myoglobin (Myo), and creatine kinase isoenzyme (CK-MB) were not used for CCS adjudication. The protocol was approved by the Ethics Committee of Yongchuan People’s Hospital of Chongqing (approval No. YCQRMYY202301KS). All participants provided signed informed consent.

The preliminary population met the following criteria: (1) age ≥18 years and diagnosed with acute stroke within 72 h of symptom onset and received treatment; (2) the AIS was confirmed by cranial computed tomography (CT) and/or magnetic resonance imaging (MRI); and (3) with clinical test including coagulation tests, cardiac biomarkers, electrocardiogram (ECG) or cardiac ultrasound examination within 24 h after admission. The participants were further excluded if they met any of the criteria: (1) intracranial hemorrhage or SAH demonstrated by CT; (2) incomplete data for echocardiograph, and/or ECG data within 48 h after admission; (3) history of cardiac disease before admission; (4) cardioembolic stroke subtype; and (5) concurrent malignant neoplasm, severe viral pneumonia, electrolyte disorders, coagulation dysfunction, severe liver, kidney, or cardiopulmonary disorder (10).

### Instruments and reagents

The blood specimens were collected by an expert phlebotomist following the international standard of Clinical Laboratory Standard Institute (CLSI) (11). For each participant, the blood was sampled into both EDTA-K2- and sodium citrate-anticoagulant tubes within 24 h of admission, from which the plasma samples were then obtained through centrifugation. Samples indicating obvious hemolysis, lipemia, or jaundice were eliminated. Single-use EDTA-K_2_ anticoagulant and sodium citrate anticoagulant vacuum blood collection tubes were purchased from Chongqing Sanfeng Medical Equipment Ltd., China.

The levels of cTnI, Myo, and CK-MB were analyzed using a UniCel DxI 800 fully automated chemiluminescence immunoassay analyzer (Beckman Coulter, United States). D-Dimer and fibrinogen (FIB) were measured using an ACL TOP 700 automatic coagulation analyzer (Werfen, Spain). B-type natriuretic peptide (BNP) was detected on an immunochromatography Q8 analyzer (Zybio Inc., China). C-reactive protein (CRP) was quantified through an i-Reader S automatic immune-analyzer (i-Reader Biotechnology Co., Ltd., China).

### Statistical analysis

Continuous variables with a normal distribution are presented as mean ± standard deviation (SD), and those with a non‑normal distribution as median (interquartile range, IQR). The independent sample Student’s *t*-tests or Mann–Whitney *U*-test was used for inter group comparisons. Pearson’s chi-square test or Fisher’s exact test was used for comparison among categorical variables. Pearson’s *r* correlation coefficients were calculated to demonstrate the linear relationship among variables. To explore and evaluate CCS-related factors, a multivariate logistic regression model was established. The Firth’s penalized logistic regression, rather than the routine logistic model, was chosen to obtain unbiased estimates of odds ratios (ORs) and confidence intervals (CIs) in small samples size and in the presence of possible separation. Age and NIHSS were treated as continuous variables; cTnI was standardized before modeling due to its narrow value range. Important variables were selected based on backward stepwise regression. Further receiver operating characteristic (ROC) analysis was used to assess the diagnostic value of the selected prediction model. The overfitting of the model was assessed and corrected using the Bootstrap method (optimism correction). A *p*-value less than 0.05 was considered statistically significant. Statistical analyses were performed on R (version 4.5.2).

## Results

### Baseline characteristics of enrolled participants

**Figure 1:**
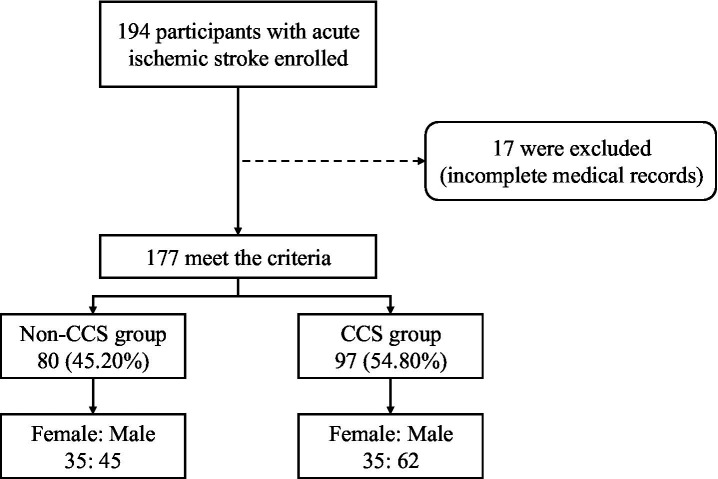
The flow chart of this study.

From March 2022 to March 2023, 194 participants who met the screening criteria were included. Furthermore, 17 were excluded due to incompleteness of medical records (Figure 1). The final cohort comprised 177 patients, of whom 97 (54.80%) belonged to the CCS group, 80 (45.20%) to the non-CCS group. Sex distribution was comparable between the CCS group [males 62 (63.92%) vs. females 35 (36.08%)] and the non-CCS group [males 45 (56.25%) vs. females 35 (43.75%)]. Patients in the CCS group were significantly older than those in the non-CCS group [median age 80 (74, 83) in the CCS group vs. 72 (66, 77) in the non-CCS group]. The lifestyle and underlying diseases were also comparable between the two groups. Besides, there were 29 (29.90%) smokers in the CCS group, compared with 22 (27.50%) in the other group. Only in the CCS group were there five (5.15%) patients with nephropathy and two (2.06%) with diabetes. The proportions of hypertension in both groups were similar. The NIHSS score was significantly higher in the CCS group (4 (1, 7)) than in the non-CCS group (1 [0, 5]). Infarct location data were collected for both groups. The most common infarct locations were the lobe(s) and basal ganglia, accounting for 44 (24.86%) and 57 (32.20%) of cases, respectively. Infarcts in the cerebellum and thalamus were least frequent, with 9 (5.08%) and 10 (5.65%) cases, respectively. In addition, no significant difference in infarct location distribution was observed between the two groups (Fisher’s exact test, *p* = 0.724).

The detailed baseline characteristics are listed in Table 1.

**Table 1:** The baseline comparisons between CCS and non-CCS groups.

Characteristic	Overall *N* = 177^a^	non-CCS *N* = 80^a^	CCS *N* = 97^a^	*p*-value^b^
Sex				0.299
Male	107 (60.45%)	45 (56.25%)	62 (63.92%)	
Female	70 (39.55%)	35 (43.75%)	35 (36.08%)	
Age	76 (69, 81)	72 (66, 77)	80 (74, 83)	<0.001
Smoke				0.726
No	126 (71.19%)	58 (72.50%)	68 (70.10%)	
Yes	51 (28.81%)	22 (27.50%)	29 (29.90%)	
Nephropathy				0.065
No	172 (97.18%)	80 (100%)	92 (94.85%)	
Yes	5 (2.82%)	0 (0%)	5 (5.15%)	
Diabetes				0.502
No	175 (98.87%)	80 (100%)	95 (97.94%)	
Yes	2 (1.13%)	0 (0%)	2 (2.06%)	
Hypertension				0.339
No	115 (64.97%)	55 (68.75%)	60 (61.86%)	
Yes	62 (35.03%)	25 (31.25%)	37 (38.14%)	
NIHSS	2 (0, 6)	1 (0, 5)	4 (1, 7)	0.008
Location				0.724
Lobe	44 (24.86%)	19 (23.75%)	25 (25.77%)	
Basal ganglia	57 (32.20%)	25 (31.25%)	32 (32.99%)	
Brain stem	20 (11.30%)	7 (8.75%)	13 (13.40%)	
Cerebellum	9 (5.08%)	4 (5.00%)	5 (5.15%)	
Thalamus	10 (5.65%)	6 (7.50%)	4 (4.12%)	
Multiple lacunar	20 (11.30%)	12 (15.00%)	8 (8.25%)	
Multiple lesions	17 (9.60%)	7 (8.75%)	10 (10.31%)	

a *n* (%); Median (Q1, Q3).

b Pearson’s Chi-squared test; Wilcoxon rank sum test; Fisher’s exact test.

### Differences and association of biomarkers between the groups

**Figure 2:**
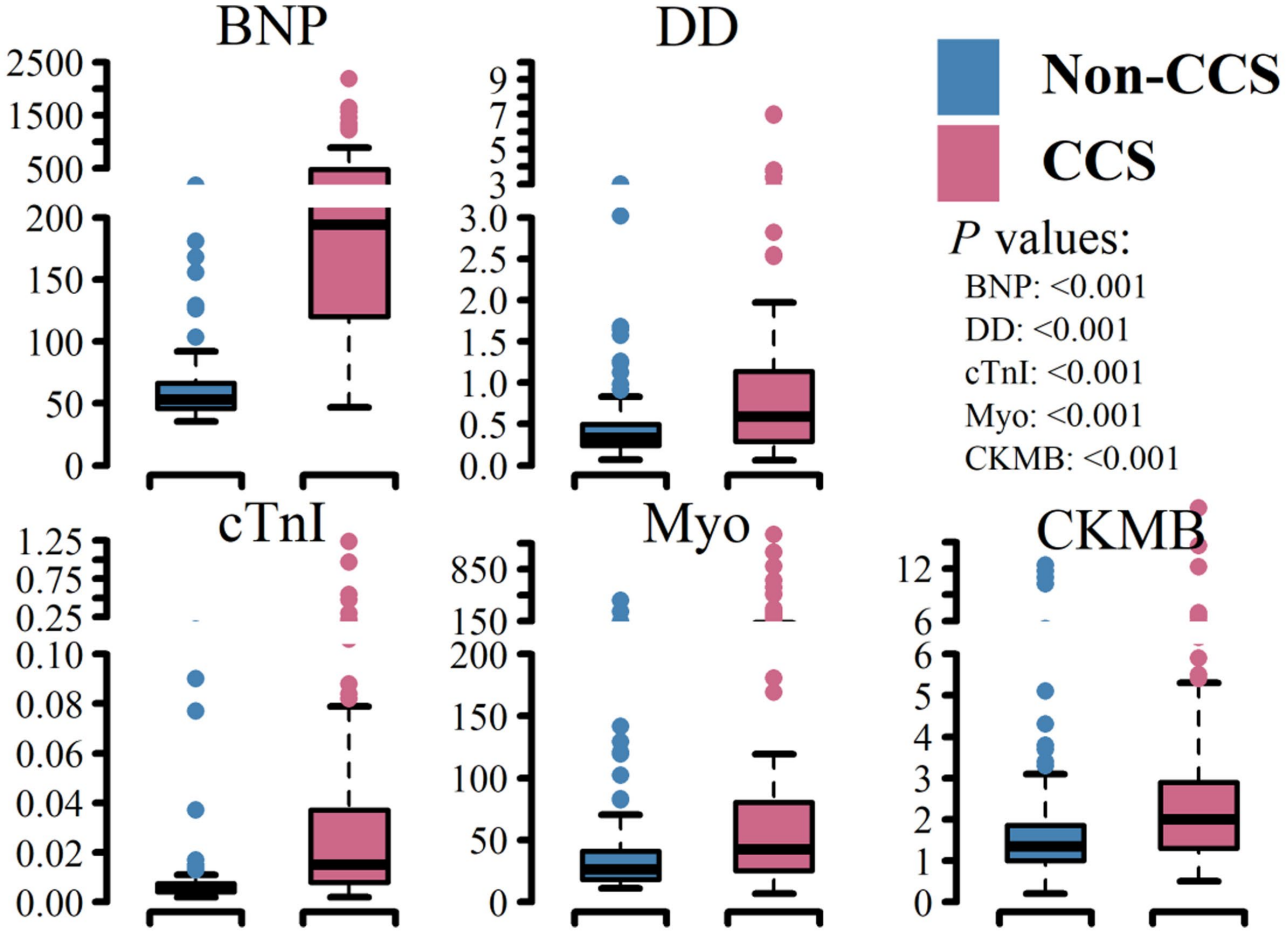
The box plot show differences of biomarkers between the groups. Comparison method: Mann–Whitney U test. All pairs are statistically significant. The *y*-axis were truncated for biomarker whose range were too large.

The levels of four cardiac biomarkers (cTnI, Myo, CK-MB, and BNP), two thrombotic markers (DD and FDP), and one inflammatory marker (CRP) were compared between the two groups. Except for FDP and CRP, which showed no significant differences, all other biomarkers were significantly elevated in the CCS group compared to the non-CCS group (all *p* < 0.001) (Figure 2 and Table 2).

**Table 2:** Comparison of biomarkers between the groups.

Characteristic	non-CCS *N* = 80^a^	CCS *N* = 97^a^	*p*-value^b^
BNP	53.14 (45.72, 66.30)	194.10 (119.98, 476.64)	<0.001
DD	0.33 (0.24, 0.50)	0.59 (0.29, 1.14)	<0.001
FDP	3.32 (2.97, 3.90)	3.39 (2.77, 4.21)	0.911
cTnI	0.01 (0.00, 0.01)	0.02 (0.01, 0.04)	<0.001
Myo	26.20 (18.20, 41.00)	42.20 (25.50, 80.20)	<0.001
CKMB	1.35 (1.00, 1.85)	2.00 (1.30, 2.90)	<0.001
CRP	2.54 (1.27, 8.96)	3.73 (1.00, 18.18)	0.803

a Median (Q1, Q3).

b Mann-Whitney U test.

**Figure 3:**
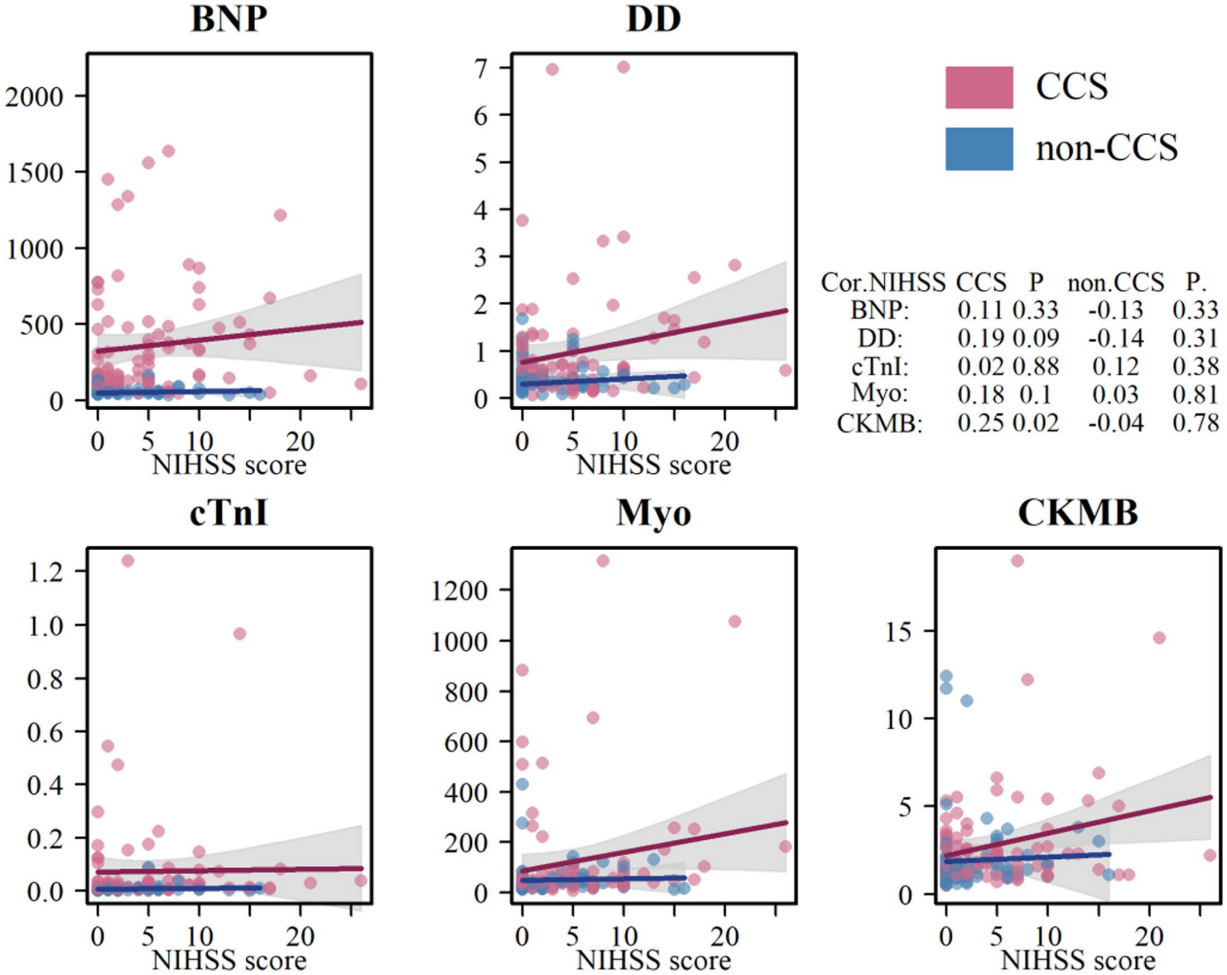
Correlations between the biomarkers and NIHSS scores. Group-specific linear regression lines with confidence bands are shown. Pearson’s correlation coefficients *r* and *p*-values for each biomarker with NIHSS score are displayed at the upper right.

The correlations between each biomarker and NIHSS score are presented in Figure 3. Distinctly different association patterns were observed between the two groups. Although only CK-MB demonstrated a statistically significant positive correlation with NIHSS score in the CCS group, BNP, DD, Myo, and CK-MB each showed an increasing trend with NIHSS scores in the CCS group, whereas their trend remained relatively flat in the non-CCS group. The statistical significance may have been attenuated by the dispersion of data.

### Risk factors for cerebral–cardiac syndrome in patients with acute ischemic stroke

To explore risk factors for the occurrence of CCS in patients with AIS, the study established a multivariable logistic regression model with adjustment for multiple clinical covariates. Given that most biomarkers differed significantly between the two groups, Firth’s penalized logistic regression was used instead of the conventional logistic model to mitigate estimation bias due to potential separation. Additionally, under its narrow range, cTnI was standardized prior to the model fitting. All other continuous variables were retained on their original scales.

First, the infarct location was not a significant contributor to the model conditioned on other variables (penalized likelihood ratio: *χ*^2^ = 0.6405, df = 6, *p* = 0.996) and was thus excluded from subsequent regression analyses.

The highest pairwise correlation coefficient among the variables was 0.681 (between CRP and FDP), and the maximum variance inflation factor (VIF) for the linear predictor was 2.125 (for FDP), indicating that multicollinearity was not a concern.

Table 3 presents the results of the model fit of conventional logistic regression and Firth’s penalized logistic regression. The primary difference between the two models lies in the coefficients of cTnI. In conventional logistic regression, despite prior standardization, cTnI yielded an odds ratio of 6.286. Following Firth’s penalization, this estimate was attenuated to 1.977.

**Table 3:** The results of multivariate logistic regressions.

(A) Comparison of results between conventional and Firth’s penalty Logistic models						
Characteristic	Conventional model			Firth’s penalty model		
	OR	95% CI	*p*-value	OR	95% CI	*p*-value
Age	1.043	0.983, 1.114	0.179	1.046	0.996, 1.098	0.074
BNP	1.036	1.021, 1.056	<0.001	1.027	1.017, 1.037	<0.001
DD	1.880	0.548, 9.756	0.442	0.910	0.509, 1.626	0.749
FDP	1.549	0.609, 4.374	0.377	1.393	0.693, 2.800	0.352
cTnI	6.286	0.847, 2,055.546	0.381	1.977	0.688, 5.680	0.206
Myo	1.005	1.000, 1.013	0.096	1.004	1.001, 1.008	0.021
CKMB	0.824	0.577, 1.116	0.227	0.901	0.715, 1.136	0.378
CRP	0.973	0.943, 0.998	0.050	0.981	0.963, 0.999	0.043
NIHSS	1.018	0.885, 1.176	0.801	1.002	0.904, 1.111	0.964

(B) The Final model of Firth’s penalty logistic optimized by backward elimination			
Characteristic	OR	95% CI	*p*-value
Age	1.047	1.003, 1.093	0.036
BNP	1.028	1.020, 1.036	<0.001
Myo	1.003	1.000, 1.006	0.048

CI, Confidence Interval; OR, Odds Ratio.

To identify important risk factors, further backward elimination was performed on the model. The final optimized model is presented in Table 3. Compared with the full model, the reduced final model demonstrated no significant difference in fitness (*χ*^2^ = 3.114, df = 6, *p* = 0.794) while retaining only three explanatory variables. In this final model, Age (OR = 1.047, *p* = 0.036), BNP (OR = 1.028, *p* < 0.001), and Myo (OR = 1.003, *p* = 0.048) were independently associated with CCS risk in AIS patients.

### Evaluation of the diagnostic model for predicting cerebral–cardiac syndrome in acute ischemic stroke patients

**Figure 4:**
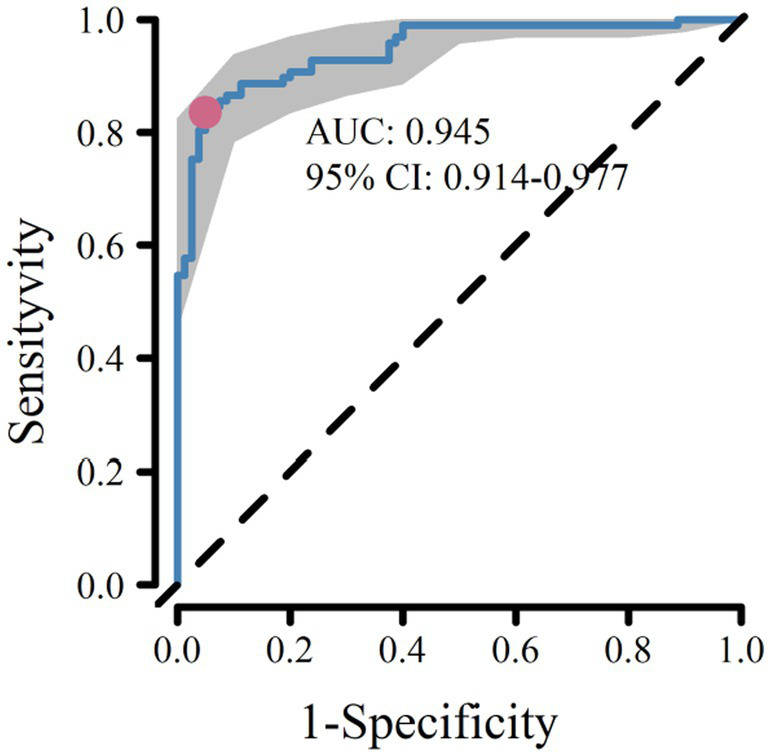
ROC curve show the performance of the predictive model.

Finally, the performance of the final model was evaluated using ROC curve analysis, as illustrated in Figure 4. The area under the curve (AUC) was 0.945 (95% confidence interval: 0.914–0.977), indicating excellent discriminatory ability and suggesting that the model can effectively predict the occurrence of CCS in patients with AIS. Furthermore, given the single-center design and relatively small sample size (*n* = 177) of this study, a bootstrap-based internal validation was performed to assess and correct potential overfitting (12). The optimism was estimated from 2000 bootstrap iterations, with a mean of 0.004. Accordingly, the optimism-corrected AUC of the final model was 0.941.

## Discussion

The key to stroke treatment lies in reducing primary injury and preventing secondary injury over time. Early identification of CCS—a common complication occurring within 72 h after acute stroke onset—is of great prognostic significance for patient treatment and outcomes (13). Based on earlier research studies, hypertension, diabetes, chronic kidney disease, and history of smoking are all risk factors for CCS (14). However, no significant between-group differences were observed for baseline characteristics of the identified risk factors in this study. The overall incidence of CCS in the study cohort was 54.80%, which aligns closely with findings from earlier studies (10, 14). Nevertheless, this incidence remains relatively high, which might be attributable to the fact that the diagnosis of CCS in this study has been mainly based on electrocardiographic changes. According to the literature (14), the incidence of post-stroke electrocardiographic abnormalities is inherently high. Different diagnostic criteria applied for CCS may account for the large variation in the reported incidence of CCS (15). Prior studies have demonstrated that stroke complicated by CCS is associated with prolonged hospital stays and increased medical expenditures (13). Thus, early detection and prevention of CCS may enhance patients’ outcomes and survival while reducing healthcare costs.

BNP is a well-established cardiac biomarker routinely used in the diagnosis and prognosis of heart failure. Secreted predominantly by ventricular myocytes, it binds to natriuretic peptide receptor-A (NPR-A) and enhances cyclic guanosine monophosphate (cGMP) production, which in turn induces natriuresis, diuresis, and vasodilation—thereby lowering blood pressure and alleviating cardiac preload and afterload (16). It also has biological functions such as inhibiting myocardial fibrosis and myocardial remodeling (17). In recent years, researchers have suggested that plasma BNP levels at admission can predict the short-term prognosis and in-hospital mortality of AIS patients (18). Zhang et al. (19) suggested that continuous BNP monitoring may offer superior diagnostic utility compared to single measurements in patients with acute cerebral infarction complicated by heart failure. In addition, natriuretic peptides such as BNP and NT-proBNP have also been shown to effectively predict covert paroxysmal atrial fibrillation after ischemic stroke (25). Such a dynamic assessment enables more timely recognition of worsening cardiac function. This is particularly important for reflecting real-time clinical changes in this complex patient population. Other cardiac biomarkers, such as cTnI, Myo, and CK-MB, are also widely used by clinical studies as specific biomarkers for the quantitative identification of myocardial injury. Guidelines from the American College of Cardiology have indicated that myocardial injury will occur frequently after AIS, and the prognosis of neurological function and hospital mortality levels are highly correlated with myocardial injury (19). Previous studies have reported that myocardial injury can be found in over 50% of AIS patients, accompanied by an increase in cardiac troponin within 48 h (20, 26). The clinical diagnostic criteria for CCS adopted in this study did not incorporate cTnI. A substantial proportion of CCS patients (82/96, 85.4%) exhibited cTnI levels that overlapped with the range observed in the non-CCS group (for CCS group: cTnI ranged from 0.002 to 1.236, *n* = 97; for the non-CCS group: 0.002–0.090, *n* = 80). This likely accounts for the exclusion of cTnI from the final multivariable model in this study. Therefore, the applicability of cTnI in CCS diagnosis should not be questioned based solely on this result.

D-Dimer is composed of two cross-linked D-D fragments of fibrin molecules, which can reflect changes in blood hypercoagulability and fibrinolysis. It is not only closely related to the risk of cardiovascular events (21), but also an independent predictive indicator of AIS (22). Earlier studies showed that D-Dimer can significantly predict various cardiovascular events, including acute myocardial infarction, readmission of heart failure, stroke, and cardiovascular death (22, 27–29). Therefore, this study investigated the relationship between clinical levels of cardiovascular-related biomarkers and CCS. The results showed that, conditioned on other cardiovascular biomarkers, BNP and Myo had higher statistical diagnostic value for stroke-complicated CCS. Furthermore, the BNP and Myo levels in the CCS group were significantly higher than those in the non-CCS group [BNP: 194.10 (119.98, 476.64) vs. 53.14 (45.72, 66.30), *p* < 0.001; Myo: 42.20 (25.50, 80.20) vs. 26.20 (18.20, 41.00), *p* < 0.001]. Although DD, cTnI, and CK-MB also exhibited significant between-group differences, only BNP and Myo remained as independent risk factors in the final model. This result may be explained by BNP and Myo being more sensitive markers for myocardial ischemia or cardiac function, and acute myocardial injury when there is severe brain function damage.

In view of the substantial intergroup differences across most biomarkers, conventional logistic regression was susceptible to OR overestimation due to variable separation (23, 24). Accordingly, the study used Firth’s penalized logistic regression—a method developed to mitigate separation bias in small-sample settings—to generate more robust effect estimates. Upon backward removal of non-contributory variables, age (OR = 1.047, *p* = 0.036), BNP (OR = 1.028, *p* < 0.001), and Myo (OR = 1.003, *p* = 0.048) were retained as independent risk factors for CCS. The discriminatory capacity of this multi-marker panel was subsequently confirmed by ROC analysis, and further supported by bootstrap internal validation, underscoring its diagnostic value for CCS detection in the AIS population.

In summary, the timely detection of CCS following stroke is clinically imperative. BNP and Myo constitute clinically valuable biomarkers for CCS. In conjunction with age, this composite model demonstrates strong diagnostic utility for CCS discrimination among AIS patients.

This study has several limitations. First, the relatively small sample size, drawn from a single center, may limit the generalizability of the findings of this study. Larger, multicenter prospective studies are warranted to further validate the diagnostic model. Second, a lack of pre-CCS blood samples precluded the development of an earlier predictive model that could enable more timely identification and intervention.

## Data Availability

The original contributions presented in the study are included in the article/supplementary material, further inquiries can be directed to the corresponding author.
